# Low Back Pain in South African Adolescent Field Hockey Players: Implications for Future Practice

**DOI:** 10.3390/jcm14103309

**Published:** 2025-05-09

**Authors:** Dale De Wit, Julian D. Pillay

**Affiliations:** 1 Department of Chiropractic, Durban University of Technology, Durban 4001, KwaZulu Natal, South Africa; daledewit@yahoo.com; 2 Faculty of Health Sciences, Durban University of Technology, Durban 4001, KwaZulu Natal, South Africa

**Keywords:** risk factors, treatment approaches, lifestyle factors, health

## Abstract

**Background:** Field hockey is a popular sport, globally. Players repeatedly perform a combination of forward flexion and rotational movements, predisposing them to pain/injury. This study aimed to determine the 3-month period prevalence and incidence (number of new cases) of low back pain (LBP) in male adolescent field hockey players, its characteristics and association with selected risk factors, and treatment approaches. **Methods:** A questionnaire-based study was administered to 112 male adolescent players, and included sections on demographics, injury characteristics and professional care sought. **Results:** Sixty-eight questionnaires were completed (67% participation rate); period prevalence of LBP was 63.2% (35.0%: at the beginning of the season, 32.4%: at mid-season, 22.1%: at end-season); incidence was 38.2%. The most common location for LBP was the middle to low back region (39.5%); the most common duration of pain was a few hours (32.6%). Most participants (79.1%) did not classify their pain as a disability; only 44.2% of participants received medical treatment. **Conclusions:** Despite focusing on a specific group of adolescents, this study revealed a comparable, yet slightly elevated, prevalence of LBP compared to earlier research predominantly involving female populations. Consequently, we recommend the development, implementation, and rigorous evaluation of targeted strategies for the prevention and management of LBP within the sport of field hockey.

## 1. Introduction

Historically, the understanding of low back pain (LBP) in adolescents posited a lower prevalence and severity compared to adult populations. However, contemporary epidemiological data indicate a concerningly elevated incidence and chronicity in adolescents, approximating adult patterns [1,2], thereby establishing it as an increasingly significant public health concern. Furthermore, longitudinal studies have identified adolescent LBP as a salient predictor of persistent LBP into adulthood [2], emphasizing the critical need for early identification and targeted interventions within this developmentally crucial period. The biomechanical demands inherent in field hockey, a globally prevalent sport integrated into numerous school curricula, present a unique etiological pathway for LBP. The fundamental motor patterns of the sport necessitate repetitive and sustained lumbar flexion coupled with concurrent axial rotation during the critical skill of stick–ball contact [3,4]. This specific kinematic profile generates substantial and potentially injurious biomechanical loading on the lumbar spine. While direct sport-specific spinal loading investigations in field hockey are emerging, biomechanical analyses of the lumbar spine under combined flexion and rotational loads consistently demonstrate augmented stress concentrations on intervertebral disks and zygapophyseal joints [5]. Moreover, the sustained semi-crouched posture adopted by field hockey players, as hypothesized by Hidalgo-García et al. (2022) [6], likely contributes to increased compressive and shear forces across the lumbar vertebral segments, thereby increasing susceptibility to pain and injury. Despite the identified biomechanical risk factors associated with field hockey and the growing recognition of adolescent LBP as a significant public health challenge, empirical research specifically examining the prevalence, characteristics, and associated risk factors of LBP in male adolescent field hockey players remains conspicuously limited, particularly within the South African and broader African epidemiological contexts. The literature predominantly focuses on female adolescent athletes, creating a critical lacuna in our understanding of this musculoskeletal condition within their male counterparts. Addressing this knowledge gap is paramount for the development of evidence-based and targeted injury prevention strategies tailored to this specific athletic demographic, aligning with broader public health initiatives aimed at promoting lifelong musculoskeletal health and mitigating the burden of chronic pain. Consequently, the objectives of this investigation were to (i) determine the 3-month period prevalence and incidence of LBP in a cohort of male adolescent field hockey players within the South African setting; (ii) characterize the clinical presentation of LBP within this population, including anatomical location, chronicity, functional disability, and healthcare-seeking behaviors; and (iii) investigate potential associations between LBP and a range of selected demographic, general health, and lifestyle-related risk factors. The study further provides data-driven recommendations for the development of targeted prevention and management strategies applicable to male adolescent field hockey players, with potential translational implications for other sports within this age stratum.

## 2. Methodology

### 2.1. Study Design and Setting

This research study was a questionnaire-based descriptive study of cross-sectional design at selected high schools within the eThekwini municipality of Durban, KwaZulu-Natal, South Africa.

### 2.2. Study Population and Sample Size

The study population included male adolescent field hockey players attending government schools and participating on an Astroturf surface hockey field. The population age range was set between 13 and 18 years of age and included those that had completed the learner assent form along with their parent/legal guardian providing consent. Four government high schools in the eThekwini municipality met the requirements for playing hockey on Astroturf. The Raosoft^®^ online software package estimated a target population of 157 participants as a minimum response rate (70%) to provide generalizability [7]. However, two of the high schools did not provide permission to conduct research, which consequently reduced the target population to 112 participants.

### 2.3. Inclusion and Exclusion Criteria

The following criteria were used to determine those eligible for inclusion in the study:

- Male adolescents (12–19 years of age) who participate in field hockey in the eThekwini municipality.
- Field hockey players who participate on an Astroturf surface.
- Participants having completed the learner assent form along with their parent/legal guardian providing consent.

The following criteria were used to determine those not eligible for inclusion into the study:

- Participants who participated in the pilot study.
- Participants whose parent/legal guardian did not provide informed consent.
- Participants who did not sign the learner assent form.
- Participants residing outside the eThekwini municipality.

### 2.4. Measurement Tool

A self-administered questionnaire adapted using a questionnaire from Goertz et al. (2012) [8] was used. The questionnaire was modified by an expert focus group after a discussion and a subsequent pilot study. The questionnaire contained sections on demographics, general characteristics, health and lifestyle characteristics, LBP patterns, treatment of LBP and the level of disability associated with LBP. The Quebec Pain Disability Scale was used from Kopec et al. (1995) [9] and Davison et al. (2002) [10] to determine the characteristics of LBP in terms of disability. The period prevalence of LBP was defined as LBP during the three-month playing season. Point prevalence was measured at the beginning of the season, mid-season, and at the end of the season. Incidence included all participants who developed LBP over the season (i.e., the number of new cases).

### 2.5. Expert Focus Group

In order for Institutional Research Ethics Committee to grant full ethical approval of the study, a focus group was used in order to validate the pre-focus group questionnaire. The focus group assessed factors such as length and layout of the questionnaire, as well as appropriateness of the questions. Additionally, it allowed for corrections to be made with regard to any mistakes identified in questions as well as the option to remove existing questions and/or add new questions in keeping with the study objectives.

The focus group consisted of the following members to validate the questionnaire:

- The researcher;
- The research supervisor/s;
- Two potential participants;
- A Master’s student who was conducting questionnaire research;
- One lecturer and researcher with experience in quantitative research.

Participants received a copy of the questionnaire and subsequently, all questions in the questionnaire were discussed and all necessary changes or suggestions were made towards a modified questionnaire (Supplementary File).

### 2.6. Data Analysis

The data were captured on a Microsoft Excel spreadsheet and statistically analyzed using IBM SPSS version 25. Descriptive statistics were presented as frequencies by percentage and n value; means and standard deviations were also used where appropriate. Categorical variables were described using frequency tables. Associations between risk factors and LBP were tested using the Pearson’s chi-square test, in comparing those with LBP versus those without. A *p*-value < 0.05 was considered statistically significant.

## 3. Results

### 3.1. Descriptive Characteristics of Participants

Of the 112 questionnaires administered, 68 participants completed and returned their questionnaires. This resulted in a participation rate of 60.7%.

The mean age of participants was 15 years (SD 1.5 years) with an age range of 13–18 years. Table 1 provides further descriptive characteristics of respondents, categorized according to whether they experienced LBP or not.

A trend was observed with the number of years playing field hockey and pain (*p* = 0.08) where those who had played for slightly longer were more likely to have experienced LBP. The most common playing position was defender with a non-case count of 9 participants and a period prevalence count of 18 participants (Total count of 27). Participants who played both the defender or midfield position had the highest period prevalence of LBP (100%), followed by individuals who only play in the defender position (66.7%).

### 3.2. Three-Month Period Prevalence, Point Prevalence, and Incidence of Low Back Pain

Table 2 presents the 3-month period prevalence, point prevalences and incidence of LBP among the participants.

The overall 3-month period prevalence of LBP during the three-month playing season was 63.2% (n = 43/68). The point prevalence at any given time was more than 20%, with some players experiencing more than one point prevalence.

### 3.3. Characteristics of Low Back Pain

**Figure 1:**
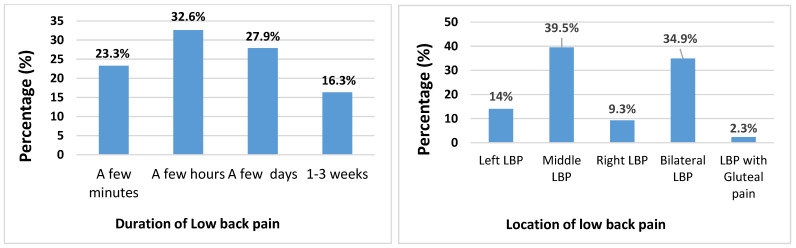
Location and duration of low back pain (N = 43).

Figure 1 provides a summary of the characteristics of LBP experienced by participants, in terms of location and duration of LBP.

Middle LBP (39.5%; n = 17/43) and bilateral LBP (34.9%; n = 15/43) were the most frequent location areas of LBP participants, with fewer participants experiencing left LBP (14%; n = 6/43), right LBP (9.3%; n = 4/43) or LBP with gluteal pain (2.3%; n = 1/43).

Most participants experienced LBP for a few hours (32.6%; n = 14/43) or a few days (27.9%; n = 12/43). Fewer respondents reported experiencing pain for a few minutes (23.3%; n = 10/43) or for more than a week (16.3%; n = 7/43).

Most players (79.1%; n = 34/43) did not experience LBP, which was classified as a disability. Furthermore, missing practices or matches due to LBP was not common and most participants did not have to adjust the way they played due to their LBP, with 93.0% (n = 40/43) of participants not missing practices or matches. In cases where practices or matches were missed (7%; n = 3/43), the maximum number of sessions missed was reported to be only two matches and this was reported by 66.7% (n = 2/43) of participants that missed playing sessions.

### 3.4. Associations Between Low Back Pain and Health and Lifestyle Factors

Table 3 summarizes the findings on health and lifestyle factors such as warming up before play, diet/nutrition, frequent hydration during training and matches, and aerobic fitness levels.

The only association found to be significant, although borderline, was hydration (*p* = 0.050).

### 3.5. Management and Treatment of Low Back Pain

Nearly half (44.2%; n = 19/43) of participants experiencing LBP received treatment from medical professionals for the LBP experienced. The participants who received treatment were mostly treated by a Chiropractor (42.1%; n = 8/19), a Physiotherapist (26.3%; n = 5/19) or a Biokineticist (15.8%; n = 3/19). Only 10.5% (n = 2/19) were treated by a medical doctor or nurse (5.3%; n = 1/19). More than half of the participants (58.1%; n = 25/43) performed self-treatment primarily through stretching (28%; n = 7/25). Other forms of self-treatment included rest, ice, and massage. A total of 53.5% (n = 23/43) of participants did not receive advice on how to manage or treat their LBP. Of the 46.5% (n = 20/43) of participants who did receive management/treatment advice for their LBP, 55% (n = 11/20) reported being advised to stretch.

## 4. Discussion

### 4.1. Prevalence and Incidence of Low Back Pain

The findings of our study reported a period prevalence of 63.2% for LBP. This is notably higher than the 33.0% prevalence reported by Van Hilst et al. (2015) [11] in young elite male field hockey players. However, our prevalence was lower than the 67.0% found by the same study [11] in their young elite female cohort. Similarly, the incidence of LBP in our study (38.2%) was lower than the 56.0% reported by Haydt et al. (2012) [12] in NCAA Division III female field hockey players. These discrepancies in prevalence and incidence may be attributed to the distinct demographic characteristics across these studies. Van Hilst et al. (2015) [11] and Haydt et al. (2012) [12] primarily focused on female participants with a mean age exceeding 18 years, thus representing a more mature athletic population compared to our adolescent cohort (mean age 15 years). Adolescents may represent a more vulnerable group for overuse injuries and pain due to the dynamic interplay between rapid skeletal growth and developing muscular strength [13]. The study by Van Hilst et al. (2015) [11] is the most demographically comparable to our own, including both male (aged 14–24 years) and female participants. However, the significantly lower LBP period prevalence (33.0%) observed in their male subgroup compared to our 63.2% could be due to the slightly older average age (18 years) of their male participants, potentially indicating a degree of musculoskeletal adaptation with increasing maturity. Furthermore, the point prevalence of LBP reported in our study at the beginning (25.0%), mid- (32.4%), and end (22.1%) of the season provides novel data, as no comparable longitudinal studies, to our knowledge, have reported these specific time-point prevalence rates in adolescent male field hockey players.

### 4.2. Characteristics of Low Back Pain

The observed predominance of mid LBP (39.5%) in the current cohort is congruent with the biomechanical stressors inherent in field hockey, wherein the frequent and prolonged lumbar flexion, often coupled with axial rotation during the execution of stick–ball contact [4], imposes substantial biomechanical loading on the intervertebral discs and adjacent spinal tissues at these segmental levels. This finding is theoretically consistent with the principles of asymmetric spinal loading, whereby the predominantly unilateral engagement necessitated by stick handling and the sustained semi-crouched posture induce uneven force distribution across the lumbar spine, potentially manifesting clinically as midline or paravertebral pain. Furthermore, the substantial proportion (34.9%) reporting bilateral lumbar pain may reflect a more generalized pattern of musculoskeletal strain resulting from the sustained postural demands and repetitive movement patterns characteristic of the sport, potentially compounded by muscular imbalances between the anterior and posterior trunk musculature, or between the dominant and non-dominant sides of the body due to the sport’s inherent asymmetry [14]. The comparatively lower prevalence of unilateral left-sided (14.0%) and right-sided (9.0%) LBP may indicate individual variability in playing mechanics, limb dominance, or specific injury mechanisms arising from asymmetrical movements during defensive maneuvers or directional changes. The limited incidence of LBP with concomitant gluteal pain (2.3%) suggests that radicular pain referral may not be a primary clinical feature of LBP within this specific adolescent field hockey population, in contrast to presentations observed in other athletic cohorts. The paucity of granular data regarding the precise anatomical distribution of LBP in prior field hockey investigations underscores the novelty of the present findings, contributing a more refined understanding of the regional presentation of LBP within this athletic discipline and informing the development of more anatomically specific preventative and rehabilitation protocols.

The observed higher prevalence of LBP on the left side (14.0%) compared to the right (9.0%) is likely attributable to the inherent asymmetry of the field hockey stroke. The rules of the sport necessitate exclusive right-handed play, resulting in a fundamental striking motion directed predominantly towards the left side of the player [15]. Biomechanical analyses utilizing electromyography (EMG) have consistently demonstrated asymmetrical activation patterns in the trunk and lower limb musculature during the field hockey swing. Specifically, the contralateral (left) oblique abdominals and lumbar paraspinal muscles exhibit greater and more sustained activation during the swing and follow-through to generate power and control the movement across the body towards the left [16]. This lateralized muscle recruitment pattern leads to increased cumulative biomechanical loading and potential for fatigue on the left side of the lumbar spine. Furthermore, the repetitive axial rotation of the torso towards the left during the striking action induces torsional stresses on the intervertebral disks and zygapophyseal joints, providing a plausible mechanism for the observed higher incidence of pain on the left side [17]. While direct, high-resolution kinematic and kinetic analyses quantifying asymmetric spinal loading specifically during the field hockey swing are an evolving area of research, the established biomechanical principles of asymmetrical athletic movements and the documented muscle activation patterns in hockey strongly support this interpretation.

### 4.3. Duration of Low Back Pain and Associated Disability/Chronicity

Most participants experienced LBP for a relatively short duration of time, as the majority experienced it for only a few hours (32.6%). This was followed by a few days (27.9%), a few minutes (23.3%) and lastly 1–3 weeks (16.3%). Collectively, 83.8% of participants experienced LBP for less than eight days. These results differ slightly from the findings of Haydt et al. (2012) [12] who showed the following duration of pain results; 1–7 days (64.0%), 8–14 days (10.0%), 15–21 days (8.0%), 22–28 days (2.0%), 1–3 months (10.0%), and lastly more than three months (4.0%). Possible explanations for the varying results may be due to the different duration of pain options that were given to participants, the difference in demographics, and lastly the sample size. Nonetheless, both studies showed that most participants experienced LBP for less than 8 days. The results of both Haydt et al. (2012) [12] and our study showed that chronic LBP was less common. Haydt et al. (2012) [12] concluded that only 4.0% of participants experienced chronic LBP, whilst the results of our study showed none of the participants to have experienced chronic LBP. Interestingly, 62.8% of participants experiencing LBP experienced the same during a previous hockey season. It is therefore evident that if participants experienced LBP during a previous season, they were more likely to develop LBP in the current/future playing season.

### 4.4. Treatment and Management

The low proportion of participants seeking professional care for their reported LBP (44.2%), despite a high level of accessibility to medical professionals within their sporting environment (88.4%), warrants further exploration into potential barriers. This discrepancy could be indicative of several interconnected factors. Firstly, access to care, while seemingly available, might be perceived as inconvenient or time consuming for young athletes balancing academic and sporting commitments. The type of medical professional available at school or training might also influence their decision to seek help; for instance, access to a general practitioner versus a sports medicine specialist could affect their perceived benefit of seeking care. Secondly, injury reporting behavior in youth sports is often influenced by a complex interplay of factors. Athletes may downplay their pain due to a desire to continue playing, fear of being seen as weak or letting their team down, or a lack of awareness regarding the potential long-term consequences of untreated LBP. The sporting culture within field hockey, and the attitudes of coaches and peers towards injury, could significantly impact a young athlete’s willingness to report and seek treatment for their pain. Finally, cultural attitudes toward pain in youth sports might contribute to this low treatment-seeking rate. A “no pain, no gain” mentality, often prevalent in competitive sports, can normalize pain and discourage athletes from seeking help for what they might perceive as a minor or expected discomfort. This is particularly relevant in demanding sports like field hockey, where physical exertion and minor injuries might be considered part of the game. The fact that a significant proportion of those who did seek care consulted a Chiropractor (42.1%) aligns with evidence-based practice that recognizes spinal manipulative therapy as a potentially beneficial short-term treatment for LBP [18]. However, the overall low uptake of professional help suggests a need to address the underlying barriers related to access, reporting behavior, and cultural norms surrounding pain in adolescent field hockey.

### 4.5. Selected Risk Factors

Our results showed no statistical significance between age, BMI, the number of matches played during the season, or the number of times played per week (during the season) and LBP. There was, however, a trend observed with the number of years playing field hockey and LBP, where those who had played for slightly longer were more likely to have experienced LBP. The results of this study support other studies which have shown that higher training volumes contribute to a high prevalence of LBP [19,20].

### 4.6. Playing Position

Due to the limited and conflicting information amongst studies conducted in field hockey, it is difficult to compare results as to what playing position experiences the highest prevalence of LBP. Sharma et al. (2012) [21] found goalkeepers to have the highest prevalence, followed by defenders and midfielders and lastly forwards. Ellapen et al. (2014) [22] found that defenders had the highest rate of LBP cases, followed by forwards, midfielders, and lastly goalkeepers. This study faced the same outcome as previous studies providing conflicting results. Participants who played both the defender or midfield position had the highest period prevalence of LBP (100.0%). Followed by defenders (66.7%), goalkeepers (62.5%), forwards (61.5%), midfielders (56.3%) and lastly midfield or forward (0%). Furthermore, a p-value was not able to be calculated due to too many small cell counts in the data. With the information gathered from previous studies and the insignificant p-value findings of this study, it is unclear as to which position experiences LBP more frequently.

### 4.7. Health and Lifestyle

Health and lifestyle investigated the following: warm up before play, diet/nutrition, frequent hydration during training and matches, and aerobic fitness levels. With regard to warming up before play, almost all participants reported that they warm up before playing (n = 67; 98.5%). It was therefore not meaningful to determine if there was an association between warming up before play and LBP prevalence in a group that was nearly 100% compliant with warming up/stretching.

With regard to hydration, participants who frequently hydrated during matches and training had a LBP prevalence of 59.7%. The participants who did not hydrate frequently during matches and training had a LBP prevalence of 100.0%. Pearson’s Chi-squared test found the results to be statistically significant (*p* = 0.050) between hydration and LBP, i.e., those individuals who did not hydrate frequently were significantly more likely to experience LBP. This finding supports the literature of Lee et al. (2017) [23], who found dehydration to increase the microdamage of muscle and the exacerbation of delayed onset muscle soreness (DOMS). Additionally, the hot and humid climate of Kwazulu-Natal may have played a part in these results. This further supports Lee et al. (2017) [23] who cautioned that individuals exercising in hot and humid weather should take frequent rehydration breaks. The borderline statistical significance indicates the need to interpret this finding with caution.

With regard to aerobic fitness level, the study used the Discovery Vitality fitness level and description classification. Participants who reported an acceptable level of fitness experienced a LBP prevalence of 69.2%. Participants who reported a good fitness level experienced a LBP prevalence of 63.3%. Lastly, participants who reported an excellent fitness level experienced a LBP prevalence of 50.0%. The results showed that participants with a lower level of aerobic fitness had a higher prevalence of LBP. However, Pearson’s Chi-squared test found the results between aerobic fitness levels and LBP not to be statistically significant.

### 4.8. Strengths of the Study

To our knowledge, this is the first field hockey study to determine the incidence and the point prevalence of LBP at the beginning of the season, mid-season, and at the end of the season in male adolescents. The study also provides information on the characteristics of LBP in terms of the location and disability as well as selected risk factors specifically relating to LBP in this select group, to provide areas in which interventions can be focused.

### 4.9. Limitations and Future Recommendations

This study specifically included four government high schools within the eThekwini municipality. Unfortunately, two of these schools declined to participate in the study, which resulted in a smaller target population. This study only included male adolescent participants. The questionnaire was administered at the end of the playing season and participants were asked to recall information about their LBP. It is possible that participants may have made errors or forgotten information over the duration of the playing season.

We recommend similar studies with a larger sample size to ensure statistical power in the analyses of sub-group variables within the sample population, for example, analyses by different playing positions, etc. We also recommend future prospective studies that will analyze variables such as hip strength, muscle imbalance, dynamic postural control, core stability, training load, etc., as potential injury risk factors, beyond those analyzed in this study.

## 5. Conclusions and Recommendations

Focusing on a specific group of male adolescent field hockey players, this study revealed a significant finding: the prevalence of LBP was comparable to, and even slightly exceeded, rates reported in earlier studies primarily involving female adolescents. This highlights the substantial impact of LBP across the adolescent field hockey population, irrespective of gender, suggesting a possible underestimation of its occurrence in males. Therefore, we urgently recommend a comprehensive and systematic effort involving the development, meticulous application, and rigorous assessment of targeted, evidence-informed strategies for both preventing the initial onset and effectively managing existing LBP within the demanding physical environment of adolescent field hockey. Future research should prioritize identifying unique risk factors associated with the sport, designing tailored exercise programs for intervention, and implementing educational initiatives aimed at athletes, coaches, and parents to reduce the incidence and consequences of LBP in this athletic group. Furthermore, longitudinal investigations are necessary to monitor the long-term efficacy of these strategies and to gain a deeper understanding of how LBP develops and progresses in adolescent field hockey players.

## Figures and Tables

**Table 1:** Descriptive characteristics of participants (N = 68).

Indicator	Prevalence	n	Mean	SD	SEM	*p*
Height (cm)	No LBP	25	170.8	10.5	2.1	0.13
	LBP	43	174.7	9.8	1.5	
Body mass (kg)	No LBP	25	62.4	9.8	2.0	0.12
	LBP	43	66.0	8.7	1.3	
Age (years)	No LBP	25	15.3	1.6	0.3	0.72
	LBP	43	15.4	1.4	0.2	
Number of matches played during the season	No LBP	25	21.7	7.5	1.5	0.58
	LBP	43	23.0	9.7	1.5	
Number of times played per week (during the season)	No LBP	25	4.6	1.1	0.23	0.5
	LBP	43	5.0	1.6	0.2	
Number of years played	No LBP	25	5.8	2.1	0.4	0.08
	LBP	43	6.8	2.1	0.3	
Playing position						
(a) Goalkeeper	No LBP	3 (37%)	-	-	-	-
	LBP	5 (62.5%)	-	-	-	-
(b) Defender	No LBP	9 (33%)	-	-	-	-
	LBP	18 (66.7%)	-	-	-	-
(c) Midfield	No LBP	7 (43%)	-	-	-	-
	LBP	9 (56.3%)	-	-	-	-
(d) Forward	No LBP	5 (38.5%)	-	-	-	-
	LBP	8 (61.3%)	-	-	-	-
(e) Defender or midfield	No LBP	0 (0.0%)	-	-	-	-
	LBP	3 (100.0%)	-	-	-	-
(f) Midfield or forward	No LBP	1 (100.0%)	-	-	-	-
	LBP	0 (0.0%)	-	-	-	-

For playing positions, no statistical estimates are provided due to the small sub-groupings. SD: Std. Deviation; SEM: Std. Error Mean; *p*: *p* Value.

**Table 2:** Prevalence and incidence of low back pain (N = 68).

	No Low Back Pain		Low Back Pain	
	Frequency (n)	Percentage (%)	Frequency (n)	Percentage (%)
3-month period prevalence	25	36.8%	43	63.2%
Incidence	42	61.8%	26	38.2%
Point prevalence beginning of season	51	75%	17	25%
Point prevalence mid-season	46	67.6%	22	32.4%
Point prevalence end of season	53	77.9%	15	22.1%

**Table 3:** Association between low back pain and health and lifestyle factors.

Risk Factor		Low Back Pain Prevalence				*p* Value
		Yes		No		
		n	%	n	%	
Warm up before play	No	1	100.0%	0	0.0%	0.186
	Yes	24	35.8%	43	64.2%	
Diet/nutrition	Very healthy	0	0.0%	2	100.0%	0.597
	Healthy	12	40.0%	18	60.0%	
	Moderately healthy	13	37.1%	22	62.9%	
	Unhealthy	0	0.0%	1	100.0%	
Frequent hydration during training and matches	No	0	0.0%	6	100.0%	0.050
	Yes	25	40.3%	37	59.7%	
Aerobic fitness levels	Excellent	6	50.0%	6	50.0%	0.520
	Good	11	36.7%	19	63.3%	
	Acceptable	8	30.8%	18	69.2%	

## Data Availability

The datasets used and/or analyzed during the current study are available from the corresponding author upon reasonable request.

## Supplementary Materials

The following supporting information can be downloaded at: https://www.mdpi.com/article/10.3390/jcm14103309/s1, File SI: Questionnaire.

## Abbreviations

LBP	Lower back pain
NCAA division III	National Collegiate Athletic Association in the United States
SD	Standard deviation
BMI	Body mass index
EBP	Evidence-based practice
DOMS	Delayed onset muscle soreness
