# Supplementary material for: Low Back Pain in South African Adolescent Field Hockey Players: Implications for Future Practice
Source: J Clin Med. 2025 May 9;14(10):3309. doi: 10.3390/jcm14103309 (PMC12112025; doi:10.3390/jcm14103309)
Supplement: Supplementary file 1 [file jcm-14-03309-s001.zip › jcm-3553886-supplementary.pdf]

File SI: Questionnaire

QUESTIONNAIRE ON THE EPIDEMIOLOGY OF LOW BACK PAIN IN MALE ADOLESCENT FIELD HOCKEY PLAYERS IN THE ETHEKWINI MUNICIPALITY.

Dear Participant,

Welcome to my research study, please fill in all questions by indicating next to the option that best applies to you. Please note that all information provided is confidential.

**To be measured by the researcher:**
**Height: ________________**
**Weight: ________________**

Section A: Demographics (please circle appropriate answers)

1. **Age?** 12yrs 13yrs 14yrs 15yrs 16yrs 17yrs 18yrs 19yrs

2. **Race? (for statistical purposes)**

   African Coloured Indian White Other

3. **What team do you play for?**

   u14A u16A 1st XI

   u14B u16B 2nd XI

4. **Do you represent one of the following teams?**

   District team KZN Schools Hockey SA Schools Hockey None

5. **Number of matches played this season?**

   (Please specify, e.g., 3) ________________________________

6. **Number of times you play hockey per week? (during the season)**

   (Please specify, e.g., 3) ________________________________

7. **How many years have you played hockey for?**

   (Please specify, e.g., 3) ________________________________

8. **What position do you play most often?**

   Goalkeeper Defender Midfield/Link Forward

9. **Do you participate in any other sports during the hockey season?**

   No Yes

   If yes, please specify: ____

Section B: General (please circle appropriate answers)

1. **What footwear do you use?**

   Running shoes   Trail shoes/cross-trainers   Hockey boots   Other

   If other, please specify: ____________________

2. **Do you use any of the following attire? (you may circle more than one option)**

   Shin pads   Ankle guard   Knee guard   None

3. **Does your attire feel comfortable when playing?**

   Yes   No

   If No, please specify:

   ____

   ____

4. **Do you warm up before playing?**

   Yes   No

Section C: Health and Lifestyle (please circle appropriate answers)

1. **Would you consider your diet to be healthy? (please circle one)**

   -Very healthy (Only eating foods such as: fruits, vegetables, high protein foods, etc.)

   -Healthy (Majority of the time eating foods such as: fruits, vegetables, high protein foods, etc.)

   -Moderately healthy (Combination of healthy and unhealth foods)

   -Unhealthy (Majority of the time eating foods high in sugar, takeaways, processed foods)

   -Very unhealthy (Only eating foods high in sugar, takeaways, processed foods, etc.)

2. **Do you hydrate frequently during training and matches?**

   Yes   No

3. **Would you consider yourself to be fit? (please circle one)**

   -Excellent (Highly trained exercisers at specific stages of their training programme)

   -Good (Training at high intensities four to six times a week)

   -Acceptable (Regularly active – a healthy level of fitness)

   -Fair (Sporadically do exercise at low intensities)

-Poor (Physically inactive)

Section D: Low back pain

This box is a definition of low back pain for your understanding.

> **What is low back pain?**
>
> **Low back pain is defined as being any pain that is located between the 12th rib and the skin fold below the buttocks/bum (gluteal muscles), which may be associated with or without leg pain (Krismer and Tulder, 2007**)
>
> **Pain may include; stiffness, tightness, dull pain, sharp pain, pain running down leg.**

If you have not experienced any low back pain during the hockey season you may ignore Section Four and Section Five and continue to answer Section F on Page 5.

1. **Have you experienced low back pain this season?** Yes No

   If you answered yes to question 4, please indicate at what point in time you experienced this pain. (you may select more than one option)

   -Beginning of season -Mid season -End of season

2. **Have you experienced low back pain during a previous hockey season?**

   Yes No

3. **When did you experience low back pain this season? (you may circle multiple options)**

   - First training session of season
   - During training sessions
   - Post-training
   - During a match
   - Post-match

4. **Can you identify the location of your low back pain?**

   Please mark with an 'X' on the suggested diagram to show the location in which you experienced or are currently experiencing low back pain.

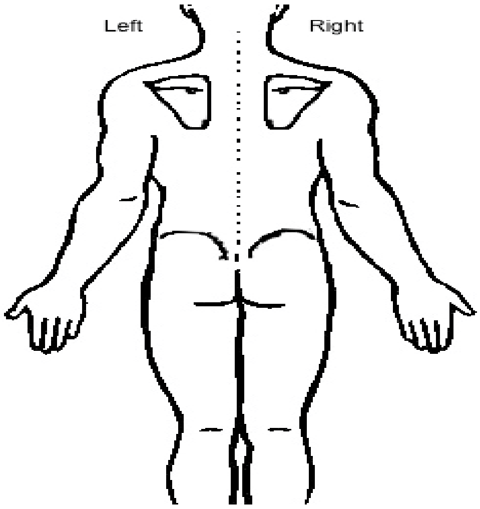

5. **How long did the pain last?**

A few minutes A few hours Days 1-3 weeks 4-12 weeks 12+ weeks

6. **Did the pain cause you to miss practice or a match?**
   Yes No
   If yes please indicated the number of practices or matches missed: _____

7. **Did the pain cause you to adjust the way you play?**
   Yes No
   If yes, please explain how:
   ____

8. **Have you experienced any injuries during the season?**
   Yes No
   If yes, please specify., e.g., sprained left ankle
   ____
   ____

Section E: Treatment (please circle appropriate answers)

1. **Did you receive medical treatment for your low back pain?** Yes No
2. **If yes, please indicate who you received treatment from:**

   -Medical Doctor -Chiropractor
   -Physiotherapist -Biokineticist
   -Nurse
   -Other: please specify ____________________________________

3. **Did you treat the pain yourself?** Yes No
   If yes, please specify (e.g., ice, stretch, rest, etc.)
   ____

4. **Are medical professionals available to you at school, matches or training?**

   Yes No

5. **If yes, please indicate which of the following:**

   -Medical Doctor -Chiropractor
   -Physiotherapist -Biokineticist
   -Nurse

-Other: please specify ___________________________________

6. **Did you receive any advice on how to manage or treat your low back pain?**
   Yes No
   If yes, please specify (e.g., ice, stretch, rest, etc.)

   ____

Section F: Disability

The Quebec Back Pain Disability Scale:

This questionnaire is about the way your back pain is affecting your daily life. People with back problems may find it difficult to perform some of their daily activities. We would like to know if you find it difficult to perform any of the activities listed below, because of your back. For each activity there is a scale of 0 to 5. Please choose one response option for each activity (do not skip any activities) and circle the corresponding number.

Did you find it difficult to perform the following activities because of your back?

	0. Not difficult at all	1. Minimally difficult	2. Somewhat difficult	3. Fairly difficult	4. Very difficult	5. Unable to do
1. Get out of bed	0	1	2	3	4	5
2. Sleep through the night	0	1	2	3	4	5
3. Turnover in bed	0	1	2	3	4	5
4. Ride in a car	0	1	2	3	4	5
5. Stand up for 20-30 minutes	0	1	2	3	4	5
6. Sit in a chair for several hours	0	1	2	3	4	5
7. Climb one flight of stairs	0	1	2	3	4	5
8. Walk a few blocks (300-400 m)	0	1	2	3	4	5
9. Walk several kilometres	0	1	2	3	4	5
10. Reach up to high shelves	0	1	2	3	4	5
11. Throw a ball	0	1	2	3	4	5
12. Run one block (about 100m)	0	1	2	3	4	5
13. Take food out of the refrigerator	0	1	2	3	4	5
14. Make your bed	0	1	2	3	4	5
15. Put on socks	0	1	2	3	4	5
16. Bend over to clean the bathtub	0	1	2	3	4	5
17. Move a chair	0	1	2	3	4	5
18. Pull or push heavy doors	0	1	2	3	4	5
19. Carry two bags of groceries	0	1	2	3	4	5
20. Lift and carry a heavy suitcase	0	1	2	3	4	5

Add the numbers for a total score: __________

THE END; THANK YOU FOR YOUR PARTICIPATION
